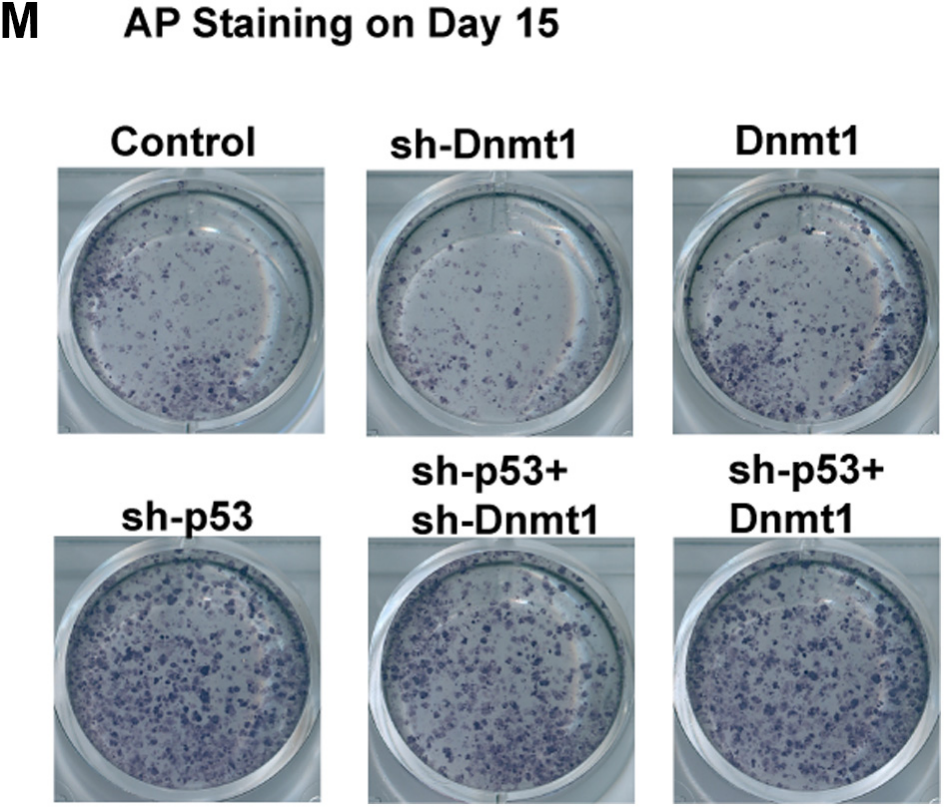# Correction: Passive DNA demethylation preferentially up-regulates pluripotency-related genes and facilitates the generation of induced pluripotent stem cells

**DOI:** 10.1016/j.jbc.2022.102566

**Published:** 2022-10-15

**Authors:** Songwei He, Hao Sun, Lilong Lin, Yixin Zhang, Jinlong Chen, Lining Liang, Yuan Li, Mengdan Zhang, Xiao Yang, Xiaoshan Wang, Fuhui Wang, Feiyan Zhu, Jiekai Chen, Duanqing Pei, Hui Zheng

The authors conclude that they inadvertently provided wrong sh-p53 and sh-p53+sh-Dnmt1 images in Figure 5M during the manuscript revision. The amended Figure 5M below shows the correct images. The corrections do not affect the results and conclusions of the article. The authors apologize for the mistake.

**Figure:**